# Morgellons disease, illuminating an undefined illness: a case series

**DOI:** 10.4076/1752-1947-3-8243

**Published:** 2009-07-01

**Authors:** William T Harvey, Robert C Bransfield, Dana E Mercer, Andrew J Wright, Rebecca M Ricchi, Mary M Leitao

**Affiliations:** 1 Preventive Medicine, Colorado Springs, CO 80949, USA; 2 Psychiatry, Red Bank, NJ, 07701, USA; 3 Veterinary Medicine, Fulton, TX, 78358, USA; 4 General Practitioner, Bolton BL1 4QR, UK; 5 Adult Medicine, USAFA, CO, 80840, USA; 6 BS (Biology), Guilderland, NY 12084, USA

## Abstract

**Introduction:**

This review of 25 consecutive patients with Morgellons disease (MD) was undertaken for two primary and extremely fundamental reasons. For semantic accuracy, there is only one "proven" MD patient: the child first given that label. The remainder of inclusive individuals adopted the label based on related descriptions from 1544 through 1884, an internet description quoted from Sir Thomas Browne (1674), or was given the label by practitioners using similar sources. Until now, there has been no formal characterization of MD from detailed examination of all body systems. Our second purpose was to differentiate MD from Delusions of Parasitosis (DP), another "informal" label that fit most of our MD patients. How we defined and how we treated these patients depended literally on factual data that would determine outcome. How they were labeled in one sense was irrelevant, except for the confusing conflict rampant in the medical community, possibly significantly skewing treatment outcomes.

**Case presentation:**

Clinical information was collected from 25 of 30 consecutive self-defined patients with Morgellons disease consisting of laboratory data, medical history and physical examination findings. Abnormalities were quantified and grouped by system, then compared and summarized, but the numbers were too small for more complex mathematical analysis. The quantification of physical and laboratory abnormalities allowed at least the creation of a practical clinical boundary, separating probable Morgellon*s* from non-Morgellons patients. All the 25 patients studied meet the most commonly used DP definitions.

**Conclusions:**

These data suggest Morgellons disease can be characterized as a physical human illness with an often-related delusional component in adults. All medical histories support that behavioral aberrancies onset only after physical symptoms. The identified abnormalities include both immune deficiency and chronic inflammatory markers that correlate strongly with immune cytokine excess. The review of 251 current NLM DP references leads us to the possibility that Morgellons disease and DP are grossly truncated labels of the same illness but with the reversal of the cause-effect order. Further, the patients' data suggest that both illnesses have an infectious origin.

## Introduction

The term "Morgellons disease" first publicly appeared on the Internet in 2002. The "index" case was the first modern case to which that label was appended: a sick child whose physical signs and symptoms were collectively unrecognized as an entity at local and regional medical facilities. As the child's illness persisted without recognition or resolution, the unaffected parent sought similar illness descriptions from historic medical references, eventually settling on "The Morgellons", a label given to childhood cases described in France in 1674 by Sir Thomas Browne [1]. His description was limited to its dermal components: hair-like extrusions and sensations of movement. Ettmuller, a physician, produced the only known drawings of The Morgellons in 1682 and considered these dermal "filaments" a parasitic infestation [1]. Numerous isolated descriptions of similar phenomena were found by Kellett from 1544 through 1884 that began as a pediatric-only illness identified by posterior torso "bristles" but also included extreme agitation, seizures, "wasting" and death (characteristics not seen in the 21^st^ century). By the early 1600s, the illness was thought to be caused by the parasite *Dracunculus* (later called *Dracontia*, then *pilaris affectio*) and "cured" by filament (agent) removal from the skin. The worm theory was considered debunked by 1715 when the esteemed microscopist, Leuvenhoeck, pronounced the bristles "inanimate". From this point forward to 1884, however, the illness shifted toward comedones, facial regions and to adults. And *Demodex folliculorum* was considered a frequent occupant of comedones, again supporting a "parasite" concept.

The name "Morgellons disease" was thus created in 2002, as a practical "place holder" because of its dermal similarity to The Morgellons described by Browne in particular (Browne's The Morgellons label evolved further over three centuries into the words, "Masquelon", then *Masclous dicti* and *Les Crinons*, shifting to Comedones after 1800). The new label also provided an alternative to differentiate a clearly, non-delusional child from many decades of clinical use of the label Delusions of Parasitosis [2]-[10]. This first set of actions would eventually raise one of the major philosophical questions of modern medicine: Does the practice of "evidence-based medicine" come from peer-reviewed literature, or from the actual patient?

Information from what was later to become the Morgellons Research Foundation (MRF) website initially provided a broader look at the believed Morgellons disease symptoms by allowing patient registrants to record their symptoms and signs. Clearly, most of the 21^st^ century patients did not fit the descriptions from 1500-1800. The MRF website provided a glimpse of the current global prevalence of those considering they are affected by this phenomenon. On January 2009, there are 13,000+ family registrants from all U.S. states and from 15 other countries. [http://www.morgellons.com/]

Given the growing use of the label Morgellons disease, characterizing the illness using verifiable clinical data seemed to be the imperative first step for clinicians, particularly to separate these patients from those two to five centuries before presentation. Until 2007, Morgellons and DP cases were often seen in isolation, interfering with credible Case Definitions. Assembling even this small number of candidate patients into a series with verified data points, should at least improved diagnosis consistency.

### Case Series Data Collection

The study protocol was simply a collection of clinical data obtained from new Morgellons disease patients at one clinic in a two-hour session between September 2006 and July 2007. Patients were not solicited but came self-diagnosed. Practice size limited the original patient number to 30 adults. The final number was 25 after filtering for consistency and data errors. Each clinical session included a detailed history, review of systems and a physical examination emphasizing dermal, neurological, endocrine and psychiatric systems. Expanded laboratory tests included a blood count (CBC), a metabolic panel (CMP) and others to evaluate major organ system function to characterize immune status, detect inflammation and search for an initial and limited number of possible infectious pathogens suggested by several veterinarians [personal communication].

Subjective screening criteria derived from earlier clinical encounters were first applied to improve patient selection consistency (Table 1). The screened data were then entered into 492 fields on each patient, 407 fields of which contained useful data points. This provided 10,175 total parameters for analysis.

**Table 1:** Initial Patient Screening Criteria

1. Patient convinced of chronic parasite infestation.
2. Primary patient illness focus must be either on dermal sensations or on "never-before-seen" material thought to be extruded from his or her body, even if this was not the most debilitating symptom or sign. The material must have been described as "fibers", "fiber-like", or "filaments".
3. Chronic pruritis (itching) must have been present for at least six months.
4. The patient must have at least two chronic, unhealed skin lesions recorded by a clinician, regardless of whether excoriation was suspected.
5. Prior diagnosis of *Delusions of Parasitosis* or *bipolar illness* would not exclude the patient as a candidate.
6. Patients experiencing delusional states common in withdrawal from drugs such as opiates and patients in organic brain states, were excluded.
7. The illness must have had a life-altering effect on the patient.
8. The patient must be an adult and had experiencing symptoms for more than six months.
9. A healthy pre-morbid period in the patient's life was acceptable.

### Case Series Initial Data Summary

The first-level summary of raw data is in the Appendix, Tables A, B and C. Categories devoid of entries had no significant findings.

#### A. Demographic Information

Male and female rates were the same for those who believed they had Morgellons disease. Age spread, geographic spread and gender neutrality were large. These data suggest Caucasian bias that did not appear in data from other countries [11].

#### B. Illness History

Rural residence or recent rural travel was common. Emotional stress was not relevant, nor was season of onset. Physical stress was a common precursor. Full onset typically required one to several months from first symptom.

#### C. Past Medical History

Prior psychiatric diagnoses in more than half. The most common diagnosis was bi-polar illness.

#### D. Social History

Significantly reduced exercise capacity was highly prevalent although non-specific as an illness marker. Recent exposure to unhygienic settings (Third world countries, sewage systems and land fills) correlated strongly with illness onset.

#### E. Systems Information from physical examination and History of Present Illness

General: Weight gain after illness onset averaged to 33 pounds. High levels of fatigue and reduced exercise capacity were eventually present in 80% of patients. Recurrent fever by thermometry was noted by 50% of patients.

Dermatological: A typical phrase used by most patients was similar to: *Extruding* and moving skin "parasites" (filaments, "fibers", "spheres") generating uncomfortable lesions. Patients stated "filament" appearance was intermittent and consistent with identifying in less than one in three patients on examination. The sensation of movement, however*,* was denied by up to 50% of those experiencing filament extrusions. About 70% had regular appearance of painful shallow skin ulcers, but most could easily separate excoriations (scratching) they had generated from spontaneous ulcers. Numerous micro-angiomas (0.5 to 3.0 mm in diameter) were found by examination on 72% of patients. This phenomenon is a known hallmark of Bartonellosis, although our patients had completely negative Bartonella serology [12,13]. Many patients noted that angiomata appearance directly paralleled illness onset and progression.

Central Nervous System: This group experienced a high rate of headaches, visual aberrancies, tinnitus, and short-term memory deficits. Emotional lability was present in more than half, typically manifest intermittently.

Cardiovascular: Patients often reported orthostatic intolerance and frequent arrhythmias (type undetermined) regardless of age. Rhythm and mild cardiac auscultation abnormalities (valvular) were commonly found on vital sign determination. Pulse was high (>72) in virtually all.

Psychiatric: Strikingly, most patients in this study (23 out of 25) had prior psychiatric diagnoses (most determined by specialists) as follows: 11 out of 25 bipolar disease; 7 out of 25 Adult ADD; 4 out of 25 Obsessive Compulsive Disorder (OCD); and 1 out of 25 Schizophrenia. Although overlap occurred in 5 cases, only the primary psychiatric diagnosis was tabulated [14]. In each case, medical records indicated that the dermal symptoms and signs preceded or occurred simultaneously with the onset of emotional signs, with an emotionally "normal" time in each patient's life prior to Morgellons disease.

Endocrine: Eight patients (32%) had prior diagnoses of Hashimoto's Thyroiditis. [The U.S. adult prevalence rate of Hashimoto's Thyroiditis is 0.56%. The rate is based on 1996 statistics: 1,490,371 adults with Hashimoto's Thyroiditis per U.S. population of 264,162,000. (Reference: Rose and Mackay, 1998, The Autoimmune Diseases, Third Edition)] Half had past evidence of Hypercalcemia (intermittent), of which 3 had definite parathyroid adenomas, later surgically excised with incomplete improvement. Fasting insulin levels were elevated in 100% who were tested for it (6 out of 25), as were CRH levels (also 6 out of 25 tested).

Other: Recurrent cough and dyspnea were common as GI and urinary symptoms, but none had a recent medically determined etiology.

#### F. Vital Signs

The most Consistent markers in this group were low core temperature and high resting heart rate, affecting all 25 patients.

#### G. Laboratory Data

CBC aberrancies were common but often intermittent. They included abnormal variable RBC indices, occasional low-grade anemia, low white cell count, and high monocyte count. Other abnormalities included low Natural Killer cell (CD56 + CD16) number and percentage, high or high-normal insulin level (in all tested) and intermittent elevation of serum calcium, globulin level, and A/G ratio. Sedimentation rate was mid-range "normal" or lower with only one ANA positive. Anti-double stranded DNA antibody level was *negative* in all tested. Occasionally IgG subclasses 1 and 3 were low. Commonly elevated markers supporting chronic inflammation included C-reactive protein and TNF-alpha. Other occasionally abnormal laboratory parameters present in chronic inflammation or infection included IFN-gamma, Homocysteine and serum Leptin. Most patients showed serologic evidence of infection (antibodies) with one or more unexpected potentially pathogenic microorganisms despite testing for only a few species.

### Data Summary

Following is the concluding summary of collected data from all patients, including pathological mechanisms suggested by the pattern(s) of these data anomalies.

Morgellons disease was often preceded by exposure to unhygienic conditions and appears first as skin abnormalities and discomfort. Illness onset appears with moderately rapid transition (weeks) from healthy to unhealthy, including "emotional discomfort". These gravid Morgellons females had an extraordinarily high miscarriage rate.

The most common physical abnormalities found in this series include: 1) Numerous "senile angiomas" on the trunk, head and limbs of many; 2) Recurrent fever; 3) Awareness of itching, crawling, stinging or biting. When present, patients describe a circadian tempo to the symptoms; some occurring solely at night. Itching of unbroken skin specifically appeared to precede all other skin symptoms; 4) Unidentified objects (called "filaments" or "granules") "extrude" uncomfortably from unbroken skin or skin lesions; 5) Painful ulcer-like concave, circular skin lesions with distinct border; 6) Excoriations adjacent-to but separate from ulcerations were common; 7) Dermal symptoms were the central focus of discomfort for most patients. 8. Multiple organ system symptoms often appeared within the first six months of illness onset. The most common systems affected were the central and peripheral nervous systems, autonomic nervous system then endocrine, cardiovascular, and pulmonary systems.

All blood pressures were low and all resting pulses were high.

Routine laboratory tests were often inconsistent and varied both positively and negatively, but within range more frequently than out of range. Common abnormalities were NK cell numbers and percentages (low), and fasting insulin levels (very high). Occasionally abnormal were RBC indices, hematocrit, WBC count (low), monocyte count (high), serum calcium (high), globulin level (high), and A/G ratio (high).

Contradictory autoimmunity data was frequently noted. Some expected inflammation tests such as sedimentation rate and Anti-double stranded DNA antibody tests were negative, while C-reactive protein and TNF-alpha were routinely high. IFN-gamma, Homocysteine and Leptin were also elevated. There was a high prevalence of transien*t* "autoimmune" diseases such as Hashimoto's Thyroiditis, hyperparathyroidism and adrenal-cortical hypofunction.

Following is a second level summary of the Morgellons data (Table 2). This is intended as a simplistic tool for clinical use.

**Table 2:** Primary Abnormal Findings

1. The large age spread, geographic spread and gender neutrality among patients suggest broad human susceptibility to the illness.
2. Rural abode or exposure to unhygienic conditions (third world travel or simply soil exposure) may be risk factors.
3. Onset rate is moderately rapid, without recognizable prodrome, commonly preceded by a healthy state.
4. Once ill, exercise capacity is significantly reduced.
5. The illness is common among family members and close associates, both related genetically and unrelated (such as spouses).
6. Most patients experience weight gain after disease onset.
7. Micro-angiomas appear rapidly on skin after illness onset in most.
8. Fever is recurrent in at least half of those affected.
9. The first illness sign may be the sudden appearance of persistent itching. Ulcerative lesions follow in some cases.
10. Once dermal symptoms begin, patients experience extrusion of unfamiliar material described variously as filamentous, "fuzz balls", black or white "flecks" or "rice grains".
11. Numerous CNS effects occur, that includes bizarre cranial nerve phenomena, anxiety and emotional sequelae. The former tend to be transient.
12. Numerous Peripheral Nervous System findings appear after illness onset. Unlike CNS effects, these are serious, permanent and progressive, and include sensory and motor nerves.
13. All Morgellons have elevated heart rate (>72 BPM) and low body temperature by oral thermometry (<97.5 degrees F).
14. Orthostatic intolerance is intermittent but common in most.
15. Most have some formally diagnosable emotional illness that begins with or becomes apparent after Morgellons disease onset.
16. Endocrine abnormality number and type is higher than background. Most common are Diabetes Type II, Hashimoto's Thyroiditis, hyperparathyroidism and adrenal hypofunction.
17. Most have elevated fasting insulin levels accompanied by elevated TNF-alpha (insulin receptor blocker) [15,16].
18. Common clinical laboratory abnormalities include:
a. RBCs have abnormal morphology. On manual examination, RBCs were non-discoid, varied in color and size.
b. Natural Killer Cell (CD 56 + CD 16) number and function are very low in most.
c. A/G ratio and globulin level are frequently elevated.
d. Sedimentation Rate and ANA are extremely low despite other common autoimmune-like conditions.
e. Elevated cytokines include: TNF-alpha, IFN-gamma, IL-6, C Reactive Protein, Homocysteine and serum Leptin.
19. Despite no fact-based Case Definition of *Delusions of Parasitosis* (DP), each of our 25 patients could have been given such an illness label as well. As pointed out by Trabert in a meta-analysis of 1,223 DP cases and others, most DP data were taken from isolated cases. Psychiatric-skewed labels were common such as "psychocutaneous disease", "acarophobia" and "monosymptomatic hypochondriasis" with no serious search for consistent physical abnormalities or microscopic parasitic agents [17].

## Conclusion

### Proposed Characterization of Morgellons Disease

The authors conclude that Morgellons disease is a multi-systemic illness that has been presumed as a delusional phenomenon for decades as its most obvious and disconcerting manifestations resembled actual (but "unverified") parasite infestation as well as various psychopathologies. However, using recent technology and even a modicum of consistently obtained physical data supports that Morgellons manifest as a skin phenomenon, an immune deficiency state and a chronic inflammatory process. Since infectious agents can initiate and maintain chronic diseases, the behavioral and other CNS manifestations here are more likely effect than cause [18]. We suggest that the Morgellons label be considered to displace any label suggesting delusion as the primary cause of this phenomenon.

The term "Morgellons disease" came into being in the 21^st^ century in an attempt to identify an illness for which no name existed. The entry of this new label into a phenomenon of extreme controversy may at first appear to further that controversy. Typical of the evolution of medical nomenclature, however, the problem may always have been with semantics; in particular the use of assumptions unsupported from failure to investigate the total physical patient [19].

The "index" case that failed to fit similar earlier diagnostic labels was seen as "different" principally because its observer looked beyond the presumed signs and symptoms of the truncated "look-alike" phenomenon labeled "Delusions of Parasitosis". Trabert's review of 1,300+ cases makes clear that only a fraction of the total signs of that presumed illness were used to create the DP diagnostic framework [14,20].

Our attempt to gather as much physical evidence on Morgellons patients as possible was based on the extremely large number of abnormal physical signs among those we evaluated earlier. We gathered as many clinical parameters as possible (within the fiscal constraints of "today's" medicine) in order to see whether the abnormalities among them were consistent and if so, whether their pattern was explanatory. The unfolding mechanism strongly suggests a chronic infectious process. The specific agent candidates will not be addressed further until evidence of their presence is available and their presence can explain the signs and symptoms we now find in all Morgellons patients.

There remains considerable work to do in collecting more data from these patients to create a credible Morgellons disease Case Definition. We submit that the same holds true for Delusions of Parasitosis patients [3]. Much of that work may be now under way by the U.S. Centers for Disease Control and Prevention (CDC) through contract with Kaiser Permanente's Northern California Division of Research. [http://www.cdc.gov/unexplaineddermopathy/] Meanwhile, the consistent abnormal findings in the data above may be used to improve clinical diagnosis and possibly initial treatment in current patients.

Perhaps of considerable importance to a journal dedicated to Case Reports is what the juxtaposition of Morgellons disease and Delusions of Parasitosis suggests. As noted by Trabert in his meta-analysis, creating a Case definition primarily from isolated cases allows uncontrolled use of assumptions that vary considerably in order to keep an unresolved conclusion constant. Where many cases are used, consistency of similar data forces a far more valid and consistent conclusion [14,20].

Until the machinery of science is in full gear and provides understanding of this phenomenon, simply "paying attention", maintaining skepticism, practicing a simple physical exam and using commercial laboratories judiciously must suffice. Once the breadth and severity of what we are encountering is understood, the resources and motivation for its solution should come. When sufficient, we anticipate the framework of several medical specialties may be modified.

## Consent

Written informed consent statements were obtained from the patients for publication of this case report. The copies of the written consent are available for review by the Editor-in-Chief of this journal.

## Competing interests

The authors declare that they do not have any competing interests.

## Authors' contributions

WH crafted the submission in its final form, including the cover letter, abstract, title page and references, drafted the summary and conclusions, and submitted the package to JMCR. RB assisted with the completion of the entire submission and was responsible for all sections addressing psychiatric elements of the illness, particularly references to *Delusions of Parasitosis*. More will follow in paper #2. DM was responsible for all references to known parasite species and all conclusion statements considering parasitosis. AW analyzed all micrographs from several fluid and tissue sources for pathogen identification and contributed data from his own pool of anonymous UK patients for context. RR obtained medical histories and systems review from all series patients and applied her FBI forensics scrutiny training to complete a second physical examination on each patient. ML is the creator of the current Morgellons label and concept, and until characterized systematically, its sole "authority". She created the Morgellons registration website, and modified this submission for consistency with the 1300+ site registrants. All authors read and approved the final manuscript.
